# Discrepancies in perceived family resilience between adolescents with chronic illness and parents: using response surface analysis to examine the relationship with adolescents’ psychological adjustment

**DOI:** 10.1186/s12888-024-05917-7

**Published:** 2024-06-27

**Authors:** Meijia Chen, Liya Ren, Hao Jiang, Yuxin Wang, Liping Zhang, Chaoqun Dong

**Affiliations:** 1 The Second Affiliated Hospital and Yuying Children’s Hospital of Wenzhou Medical University, Wenzhou, 325027 China; 2 School of Nursing, Wenzhou Medical University, University Town, Chashan, Wenzhou, 325035 China; 3 Clinical Skills Center, The Second Affiliated Hospital and Yuying Children’s Hospital of Wenzhou Medical University, 109 Xueyuan West Road, Wenzhou, 325027 China

**Keywords:** Parent-adolescent discrepancies, Adolescents with chronic illness, Psychological adaptation of adolescents, Family resilience, Response surface analysis

## Abstract

**Background:**

This study aimed to explore discrepancies in adolescents with chronic illness and their parents’ perceptions of family resilience, as well as the relationship between these differences and the psychological adjustment of adolescents with chronic illness.

**Methods:**

A cross-sectional study was conducted. A total of 264 dyads of parents (77.7% mothers, mean age 41.60 years, SD = 6.17) and adolescents (48.5% girls, mean age 12.68 years, SD = 2.11) with chronic illness were recruited through convenience sampling from three children’s hospitals in Wenzhou, Hangzhou, and Shanghai, China between June 2022 and May 2023. The Chinese version of the Family Resilience Scale and the Psychological Adjustment Scale, which are commonly used measures with good reliability and validity, were employed to assess family resilience and psychological adaption, respectively. The data were analyzed using polynomial regression and response surface analysis.

**Results:**

Adolescents with chronic illness reported higher family resilience than their parents (t=-2.80, *p* < 0.05). The correlations between family resilience and adolescents’ psychological adjustment reported by the adolescents (*r* = 0.45–0.48) were higher than parents (*r* = 0.18–0.23). In the line of congruence, there were positive linear (a1 = 1.09–1.60, *p* < 0.001) and curvilinear (a2=-1.38∼-0.72, *p* < 0.05) associations between convergent family resilience and adolescents’ psychological adjustment. In the line of incongruence, when adolescents reported lower family resilience than parents, adolescents had a lower level of psychological adjustment (a3=-1.02∼-0.45, *p* < 0.05). Adolescents’ sociability decreased when the perceived family resilience of parent-adolescent dyads converged (a4 = 1.36, *p* < 0.01).

**Conclusion:**

The findings highlighted the importance of considering the discrepancies and congruence of family resilience in the parent-child dyads when developing interventions to improve the psychological adjustment of adolescents with chronic illness. Interventions aimed at strengthening family communication to foster the convergence of perceptions of family resilience in parent-adolescent dyads were warranted.

## Introduction

Chronic diseases are defined as illnesses diagnosed based on the medical scientific knowledge that occur persistently or are expected to occur for more than three months or recur more than three times in a year [1]. The prevalence of chronic illness among adolescents continues to increase yearly. Owing to advancements in medicine and the continuous improvement in survival rates, the majority of adolescents (90%) with chronic diseases will survive into older adulthood, and this rate will continue to increase [2, 3]. Adolescence is also a crucial period for rapid physical, cognitive, social, and emotional development and has important behavioral patterns that can affect the whole person’s life [4, 5], hence it is more vital to pay attention to the mental health of adolescents compared to children or adults [4]. Strenuous treatment and the prolonged uncertainty of chronic illnesses, as well as social isolation and changes in appearance caused by disease treatment, lead to psychological distress among adolescents [6]. A previous study noted that adolescents with chronic diseases had a 51% higher adjusted risk of mental health problems than those without chronic physical diseases [7]. A recent systematic review also indicated that the prevalence of anxiety disorders in adolescents with chronic illness is higher than that in the general population [8]. However, researchers have found that many children and adolescents who have experienced trauma or adversity can adapt positively to the stress of illness in their daily lives and show good psychological adjustment [9].

The family is an important place for adolescents’ socialization processes and a significant social-ecological system that affects their psychological development [10]. Family resilience has become a focus of research as research has deepened and positive perspective studies have emerged [11]. Family resilience refers to the ability to rebound from adversity and become stronger and more resourceful [12]. Walsh’s family resilience framework highlights that family resilience comprises shared family faith systems, organizational patterns, and communication or problem-solving processes [13]. Resilient families establish support networks and foster environments conducive to patients [14]. For example, Qiu et al [15] found family resilience may influence children’s psychological adjustment through authoritative parenting. Wei [16] highlighted that families with high resilience are characterized by positive family relationships and less family conflict, which can contribute to the psychological adjustment of adolescents with chronic illness by reducing parents’ caregiver burden. Song [17] indicated that cohesive family relationships in resilient families can serve as a buffer against the risks experienced by adolescents with chronic illness by facilitating personal problem-solving and coping strategies. In conclusion, family resilience is the accumulation of protective factors in the lives of family members, as well as the positive response of an individual or family to an unfavorable situation [17]. Therefore, family resilience is essential to preserve the mental health of family members.

As the focus of research has shifted to information discrepancies in the family domain, researchers have explored the divergence in how parents and adolescents view the family process [18]. Findings show that parents and adolescents frequently disagree with various family processes, including their perceptions of family conflict and family routines [19], parental emotional socialization [20], and the quality of family communication [21]. According to Fan [20], although adolescents and parents have convergent perceptions of parental affective socialization, approximately 35% of parents and adolescents differ on the supportive parental affective socialization dimension. Reyes’ study indicated that discrepancies can provide incrementally more valuable information than even the information itself provides [22]. Discrepancies in parent-adolescent dyads can provide additional information that is unavailable in one-sided reports by either the parent or adolescent [23]. In addition, utilizing discrepancies in parent and adolescent reports can improve our understanding of psychopathology, leading to better assessment and treatment of adolescents [22]. Therefore, they have a major clinical significance and practical value. However, research on parent and adolescent family differences has not been conducted in the field of positive psychology.

Nevertheless, most existing studies on family resilience and the psychological adjustment of adolescents with chronic illness are limited to a single perspective [10, 24], ignoring the importance of parent-adolescent discrepancies in explaining the association between these two variables. Among the very few studies that investigated parent-adolescent discrepancies, the majority focused on healthy adolescents [20, 25], with few reports of adolescents with chronic illness. Rescorla reported that the extent and implications of parent-adolescent discrepancies may vary with cultural context [26]. Under the influence of traditional Confucian culture, parent-adolescent discrepancies in perceived family functioning may be exacerbated by hierarchical decision-making and insufficient emotional expression within the Chinese family, compared to Western families [27]. Furthermore, the study results in the Western context showed that adolescents had more negative views of their families than their parents and that a strong sense of filial obligation in Chinese culture may motivate adolescents to fulfill more family responsibilities and hold more positive views [27]. However, current discrepancies regarding parents’ and adolescents’ perceptions of their family environment focus primarily on Western cultural contexts [19], and few studies have focused on Eastern cultural contexts. Therefore, it is of great significance to discuss the above issues in Chinese society to enrich research in this field.

The Operational Triad Model is mainly used to explain the correlation between two reporters’ perceptions of the same “situation” and can be categorized into three types: converging operations(two reporters’ perceptions are the same), diverging operations(two reporters’ perceptions are inconsistent but there is a systematic relationship), compensating operations(two reporters’ perceptions are inconsistent and no systematic relationship exists) [28]. These three different correlations between the two reporters could have varying associations with the mental health of the reporter [28]. Therefore, the convergence and divergence between reports from parent and adolescent accounts may be associated with psychopathological symptoms in adolescents. Parent-adolescent dyads’ convergent and divergent views on family functions and environments reflect their different perspectives on the interactions and relationships between parents and children [19, 29]. The perceived congruence between parents and adolescents may be protective or beneficial, as studies have found that consistency between adolescents and parents perceived positive parent-child communication was associated with the lowest levels of adolescent depressive symptoms [30]. In contrast, discrepancies in parent and adolescent perspectives may represent maladaptive family processes [19] and should be considered a potential risk factor for adolescent psychological adjustment [28]. Empirical studies have shown that discrepancies in adolescents’ and parents’ perceptions of family functioning, which can lead to poor communication and increased conflict between adolescents and parents [31], are associated with poorer psychological adjustment reported by adolescents [19]. In addition, multiple studies have demonstrated that parental overestimation of the environment predicts poorer psychological adjustment in adolescents [25, 32, 33]. Conversely, the more favorable perception of adolescents about their family environment compared to their parents may contribute to their greater social adjustment [20]. Hence, the direction of perceived discrepancies between adolescents and their parents should also be considered when studying the psychological adaptation of adolescents with chronic illness.

The vast majority of prior studies in this area assessed discrepancies with difference scores [34](i.e., the absolute value of the difference between the two measured variables). However, there are numerous limitations in using difference scores to investigate informant discrepancy-related hypotheses. This leads to many methodological problems, including reduced reliability and validity [35]. Furthermore, the relationship between the predictors and outcome variables is oversimplified [36]. For instance, combining two different pieces of information into individual messages leads to a loss of valuable information, such as the extent to which each set of individual messages contributes to an outcome [37].

Polynomial regression and response surface analysis (RSA) have been used to test hypotheses on the effects of person-group similarity, dyadic similarity, person-environment fit, and self-other agreement and have been proven to be reliable and informative approaches for studying (in)congruence effects [37, 38]. The ability of polynomial regression to independently predict the independent predictive power of each perspective also allows for a more nuanced understanding of the relevance of different forms of consistency and inconsistency in studying outcomes within a single model. In particular, by using RSA, a three-dimensional approach, one can assess and visualize the relationship between various types of (in)congruence and the outcome variable [39], which has been widely employed in the field of organizational behaviors and psychological research [39]. Their results underscore the potential of using RSA to understand the implications of congruence and incongruence in views of family dynamics for adolescent adjustment.

## The current study

This study sought to investigate how the convergence and divergence of perceived family resilience between adolescents and parents are associated with psychological adjustment in Chinese families of adolescents with chronic illness. We hypothesized that (1) parent-adolescent convergence of high levels of family resilience would be related to an increase in psychological adjustment; (2) when adolescents’ reports of family resilience exceeded their parents’ reports, they would be more likely to report higher psychological adjustment.

## Methods

### Participants

A convenience sampling method was used in this cross-sectional study to administer a questionnaire survey to adolescents with chronic diseases and their parents in three pediatric hospitals in Wenzhou, Hangzhou, and Shanghai City, China, between June 2022 and May 2023. The inclusion criteria for adolescents with chronic diseases were as follows: (1) aged between 10 and 19 years [40] and (2) having a clear diagnosis of one of the following chronic diseases: leukemia, diabetes mellitus, chronic kidney disease, rheumatologic diseases, and chronic gastritis [1, 41]. The exclusion criteria were as follows: (1) mental illness or severe intellectual disability, (2) inability to communicate verbally or complete the questionnaire, and (3) in the terminal stage of the disease. To minimize this burden, only one parent participated in the survey. The inclusion criteria for parents were as follows: (1) parent of adolescents with chronic illness, (2) age above 18 years, (3) self-reported as being a primary caregiver for adolescents. Parents with impaired cognitive function or inability to communicate verbally or complete the questionnaire were excluded.

Using G*power to compute sample size, we calculated 80% power to detect a change in R^2^ when going from two main effects to the full polynomial model (five predictors). As such, researchers should aim for 485 observations (that is 243 dyads) to detect a small effect (f^2^ = 0.02) [42]. In the present study, a total of 289 dyads of eligible adolescents and their parents were approached, and sixteen dyads refused due to scheduling conflicts and privacy concerns. Of the 283 dyads who agreed to participate, nine dyads were excluded due to incomplete answers, resulting in a valid sample size of 264 (96.7%) dyads.

### Procedure

This study was approved by the Institutional Ethics Committees of the Second Affiliated Hospital and Yuying Children’s Hospital of Wenzhou Medical University (Approval No 2022-K-124-01). The study was conducted in accordance with the Declaration of Helsinki. A member of the research team identified potentially eligible participants by reviewing their medical records according to the inclusion criteria and exclusion criteria. A trained investigator approached the potential adolescents and their parents, either in the pediatric inpatient section of the hospital or the pediatric outpatient waiting area. The researcher explained the purpose and other details of the study to the participants, including the principles of confidentiality and anonymity. All participants signed an informed consent form before the questionnaire survey. According to the General Principles of Civil Law in China, adolescents over 16 years of age are regarded as persons with full capacity for civil behavior, and the informed consent form signed by the subject himself/herself is legally valid [43]. If adolescents with chronic illness were under 16 years of age, informed consent was also obtained from their parents. Parents were asked to complete general demographic information and the Family Resilience Scale, and adolescents were asked to complete the Family Resilience Scale and Psychological Adjustment Scale, which lasted approximately 20 to 30 min.

### Measure

#### Sociodemographic questionnaire

The sociodemographic characteristics of the adolescents included age, sex, educational level, family type, and chronic disease type. Parents’ characteristics included age, sex, educational level, employment status, and religion.

#### Family resilience

The Chinese version of the Family Resilience Scale (C-FRAS ) [44] was used to measure the perceived family resilience of both parents and adolescents. This 44-item scale includes four dimensions: family communication problem-solving, utilizing social and economic resources, maintaining positive attitudes, and giving positive meaning to adversity. Each item is rated on a 4-point Likert scale ranging from 1 (strongly disagree) to 4 (strongly agree), with higher scores indicating higher levels of family resilience. The Chinese version of the C-FRAS, which was widely used in previous studies [15, 45], was reported to have strong reliability and validity, with good internal consistency (Cronbach’s a = 0.96) and construct validity (a cumulative variance contribution of the four factors of 61.77%) [44]. In the present study, Cronbach’s alpha on the C-FRAS scale was 0.95 for parents and 0.96 for adolescents, respectively.

#### Adolescent psychological adjustment

The Chinese version of the Psychological Adjustment Scale [46] was employed to measure the psychological adjustment of adolescents with chronic illnesses. This is a 20-item scale composed of four subscales: coping skills (four items), self-improvement (six items), socialization (five items), and psychological development (five items). The adolescents were asked to rate on a 5-point Likert scale ranging from 1 (never) to 5(nearly always). Higher scores indicate better psychological adaptation. The original study reported an acceptable internal consistency (Cronbach’s a = 0.90) and construct validity (a cumulative contribution of the four factors of 55.99%) [46]. In this study, the Cronbach’s alpha of the total scale was 0.95.

### Analytical approach

Statistical analyses were conducted using the Statistical Package for the Social Sciences version 24 (SPSS, New York, NY, USA). according to the steps of Shanock et al. [37], the relationship between parent-adolescent perceived congruence and incongruence and adolescent psychological adjustment was explored using polynomial regression and response surface analysis. First, descriptive information on the discrepancies in perceived family resilience by parents-adolescent dyads was computed (Family resilience was converted into standardized scores, and the parent-reported standardized family resilience score half a standard deviation above or below the adolescent-reported standardized family resilience score was considered to have a discrepancy [47]). Then, the predictors (family resilience reported by parents and adolescents) were centralized around their scale midpoint, which helped to interpret and reduce the potential for multicollinearity. Next, three new variables were created: the square of the centered parent-reported family resilience, the cross product of the centered parent-reported and adolescent-reported family resilience, and the square of the centered adolescent-reported family resilience. Finally, polynomial regression analysis was used to examine whether the convergence and divergence in the family resilience reported by adolescents and their parents were related to the psychological adjustment of adolescents. The equation for the polynomial regression model is: Z = *b*0 + *b*1*X* + *b*2*Y* + *b*3*X*^2^ + *b*4*XY* + *b*5*Y*^2^ + e. b0 represents the intercept; b1 and b3 represent the linear effect and quadratic effect of the parent-report family resilience(X), respectively; b2 and b5 represent the linear effect and quadratic effect of the adolescent-reported family resilience (Y), respectively; b4 is the interaction term between the adolescent-reported and the parent-reported family resilience. These regression coefficients were mainly used to generate response surface models, which were primarily used to visualize the results of the polynomial regression.

The response surface analysis calculated four coefficients (a1-a4) based on regression analysis results. According to Shanock et al [37], the slope of the line of congruence (LOC) is described by parameter a1 (a1 = b1 + b2), while the form of the LOC is indicated by parameter a2(a2 = b3 + b4 + b5), which can either be linear (if a2 = 0) or curvilinear (if a2 ≠ 0). Again, the coefficient a3(a3 = b1 - b2) describes the slope of the line of incongruence (LOIC), and a4(a4 = b3 - b4 + b5) indicates whether the LOIC is linear (if a4 = 0) or curvilinear (if a4 ≠ 0).

## Result

### Descriptive statistics

The total sample consisted of 264 parent-adolescent dyads. The adolescents (48.5% girls) were, on average, 12.68 years old (SD = 2.11, range = 10–18 years), with 45.5% (*n* = 120) studying in primary school, 39.8% (*n* = 105) in middle school and 14.8% (*n* = 39) in high school. The majority of adolescents lived in a core family (61.0%, *n* = 161), 35.2% (*n* = 93) in an extended family, and 3.8% (*n* = 10) in a single-parent family. Regarding the types of chronic diseases, 91 (34.5%) adolescents were diagnosed with rheumatic diseases, 80 (30.3) with renal system diseases, 46 (17.4%) with endocrine system diseases, 45(17.0%) with hematological system diseases, and two (0.8%) with digestive system diseases. The parents (77.7% mothers) were, on average, 41.60 years old (SD = 6.17, range = 30–60 years), and 29.2% (*n* = 77) of the parents had religious beliefs. In terms of education level, 61(23.1%) parents reported an educational level of primary school or lower, 99 (37.5%) had completed middle school, 45 (17%) had completed high school, and 59(22.3%) had obtained a college degree or higher. Regarding employment status, 116 (43.9%) had full-time jobs, 39 (14.8%) had part-time jobs, and 109 (41.3%) were unemployed.

Table 1 shows the differences in family resilience reported by adolescents and their parents in terms of the general demographic information and chronic disease types. There were significant differences in parent-reported family resilience in terms of religiosity, parent’s education level, and adolescent gender(*p* < 0.05), and there was no significant difference in adolescent-reported family resilience. The family resilience did not differ significantly across different types of chronic illness, as reported by either adolescents or parents.

**Table 1:** Demographic differences in family resilience

	*N*(%)	Parents			Adolescents		
		x ± s	t/F	*p*	x ± s	t/F	*p*
**Adolescent gender**			-2.187^a^	0.03		-0.906^a^	0.366
Male	136(51.5%)	128.71 ± 12.43			132.65 ± 16.47		
Female	128(48.5%)	132.31 ± 14.34			134.59 ± 18.44		
**Highest level of education(adolescent)**			0.362	0.697		1.285	0.278
Primary school	120(45.5%)	130.36 ± 14.43			134.33 ± 17.65		
Middle school	105(39.8%)	131.11 ± 13.29			134.28 ± 17.46		
High school	**39(14.8%)**	128.97 ± 10.95			129.46 ± 16.63		
**Family classification**			0.496	0.609		1.263	0.285
Core family	161(61.0%)	130.97 ± 14.10			132.68 ± 17.00		
Extended family	93(35.2%)	129.92 ± 12.54			135.68 ± 18.24		
Single-parent family	10(3.8%)	127.10 ± 12.40			128.80 ± 16.36		
**parent gender**			-0.501^a^	0.617		-0.489^a^	0.625
Father	59(22.3%)	129.68 ± 14.04			132.61 ± 16.65		
Mather	205(77.7%)	130.68 ± 13.35			133.87 ± 17.70		
**Religious beliefs**			1.999^a^	0.047		1.615^a^	0.107
Yes	77(29.2%)	127.88 ± 12.65			130.90 ± 16.62		
No	187(70.8%)	131.51 ± 13.71			134.70 ± 17.70		
**Highest level of education(parent)**			3.153	0.025		0.766	0.515
Primary school and below^①^	61(23.1%)	126.20 ± 13.78	①**<**③		132.75 ± 14.96		
Middle school^②^	99(37.5%)	130.71 ± 12.71			132.07 ± 17.78		
High school^③^	45(17.0%)	133.40 ± 13.28			133.96 ± 15.87		
College degree or higher^④^	59(22.3%)	132.19 ± 13.89			136.73 ± 20.21		
**Employment**			0.375	0.688		0.061	0.941
Full-time	116(43.9%)	131.09 ± 11.93			133.94 ± 18.24		
Part-time job	39(14.8%)	130.97 ± 16.85			133.79 ± 18.32		
Unemployed or on leave	109(41.3%)	129.60 ± 13.80			133.15 ± 17.45		
**Disorders**			0.323	0.862		0.475	0.754
Rheumatic system diseases	91(34.5%)	131.63 ± 12.62			131.79 ± 17.29		
Endocrine system diseases	46(17.4%)	129.20 ± 13.15			133.30 ± 19.28		
Renal system diseases	80(30.3%)	130.30 ± 15.55			134.78 ± 16.86		
Hematological system diseases	45(17.0%)	129.78 ± 12.03			135.18 ± 17.06		
Digestive system diseases	2(0.8%)	127.50 ± 3.54			139.00 ± 24.04		

a for t-tests, and F-tests for the rest

Table 2 shows the means and standard deviations of family resilience reported by adolescents and their parents as well as bivariate correlations with adolescents’ psychological adjustment. Adolescents with chronic illness reported a higher level of family resilience than their parents [t =-2.80, *p* < 0.05]. Approximately 58.4% of the adolescents and parents had discrepant perceptions of family resilience. Specifically, 31.1% of parents reported higher levels of family resilience, 41.7% of parents and adolescents reported similar levels of family resilience (i.e., the discrepancies between the standardized scores were less than half the standard deviation [37]), and 27.3% of adolescents reported higher levels of family resilience. This indicates discrepancies in family resilience between parents and adolescents.

**Table 2:** Descriptive statistics for family resilience variables and correlations with adolescent psychological adjustment

	Descriptive		1	2	3	4	5
	Mean	SD					
1. Parent-reported family resilience	2.96	0.31					
2. Adolescent-reported family resilience	3.04	0.40	0.33^***^				
3. Coping Ability	3.52	0.78	0.18^**^	0.45^***^			
4. Self-improvement	3.51	0.79	0.23^***^	0.48^***^	0.72^***^		
5. Sociability	3.62	0.78	0.20^**^	0.45^***^	0.64^***^	0.68^***^	
6. Psychological Development	3.38	0.85	0.19^**^	0.47^***^	0.61^***^	0.76^***^	0.71^***^

*p < 0.05, **p < 0.01, ***p < 0.001

Correlational analyses indicated that adolescent-reported and parent-reported family resilience were positively correlated (*r* = 0.33, *p* < 0.01) Additionally, both parent-reported and adolescent-reported family resilience were positively correlated with the four subscales of adolescents’ psychological adjustment. Parents-reported family resilience had a low correlation with adolescents’ psychological adjustment (*r* = 0.18–0.23), while adolescents-reported family resilience had a moderate correlation with adolescents’ psychological adjustment (*r* = 0.45–0.48), see Table 2.

### Polynomial regression and RSA effects on adolescent psychological adjustment

**Fig. 1:**
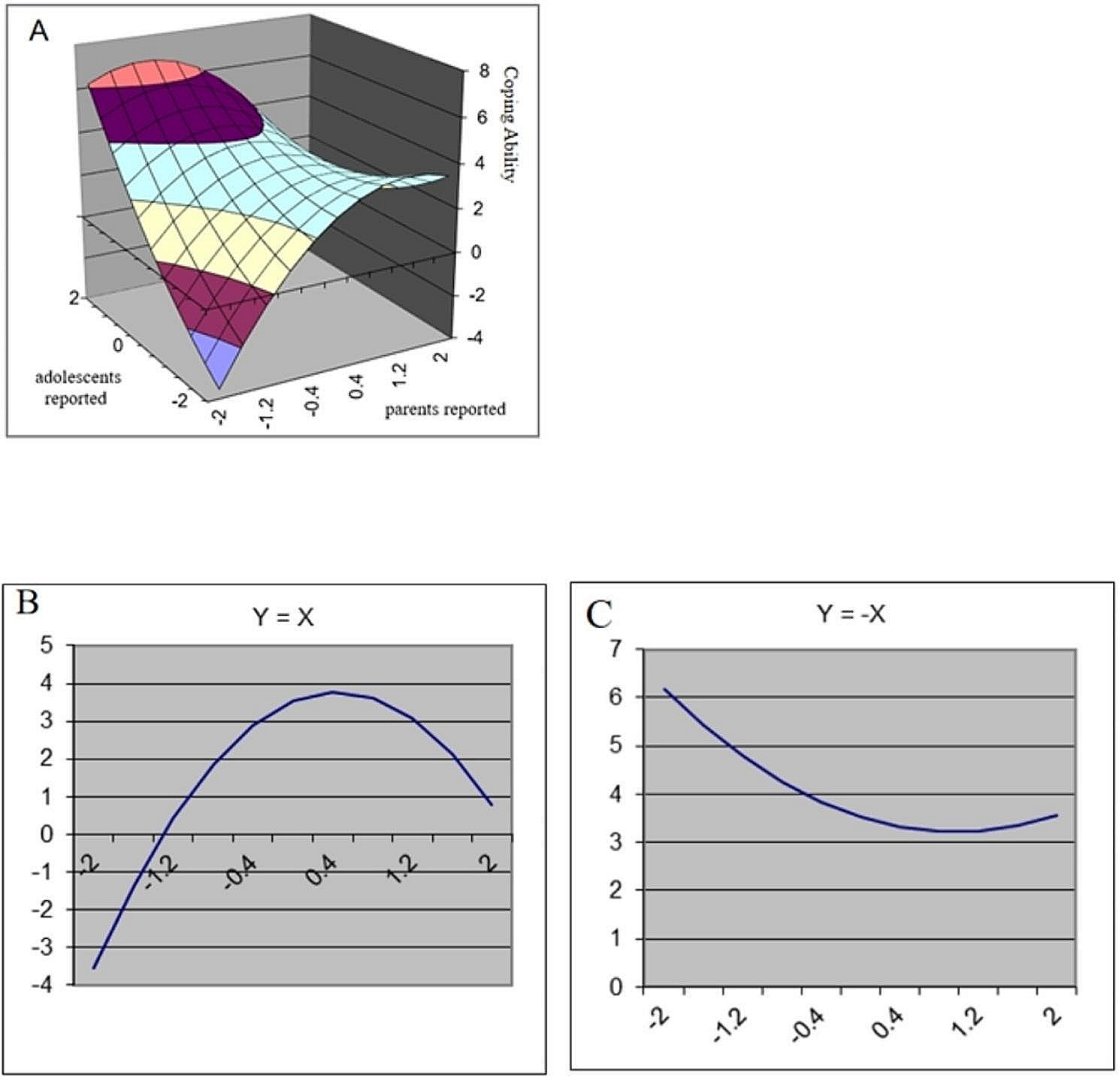
Response Surface Analysis plots for adolescents’ coping ability

**Fig. 2:**
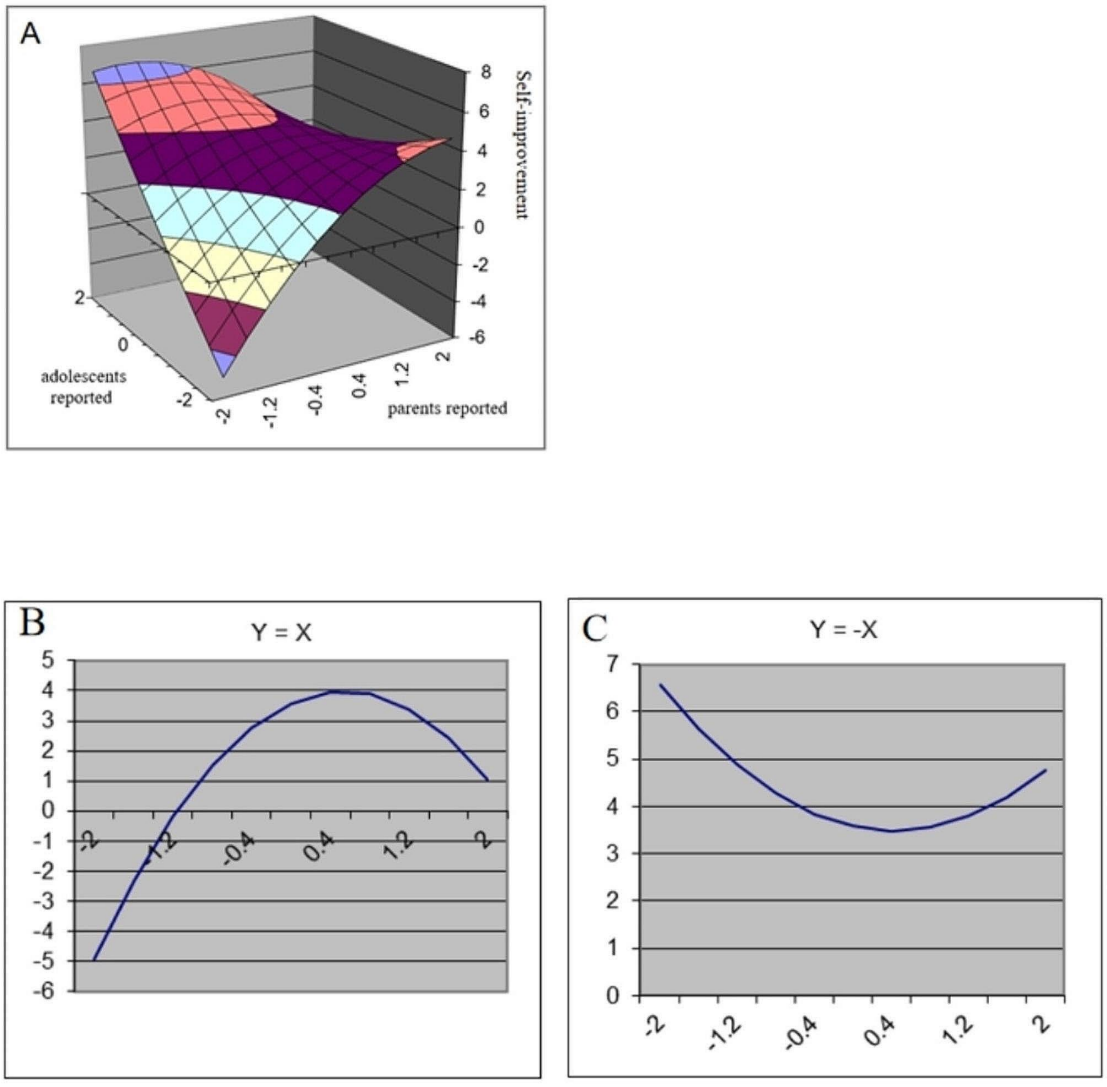
Response Surface Analysis plots for adolescents’ self-improvement

**Fig. 3:**
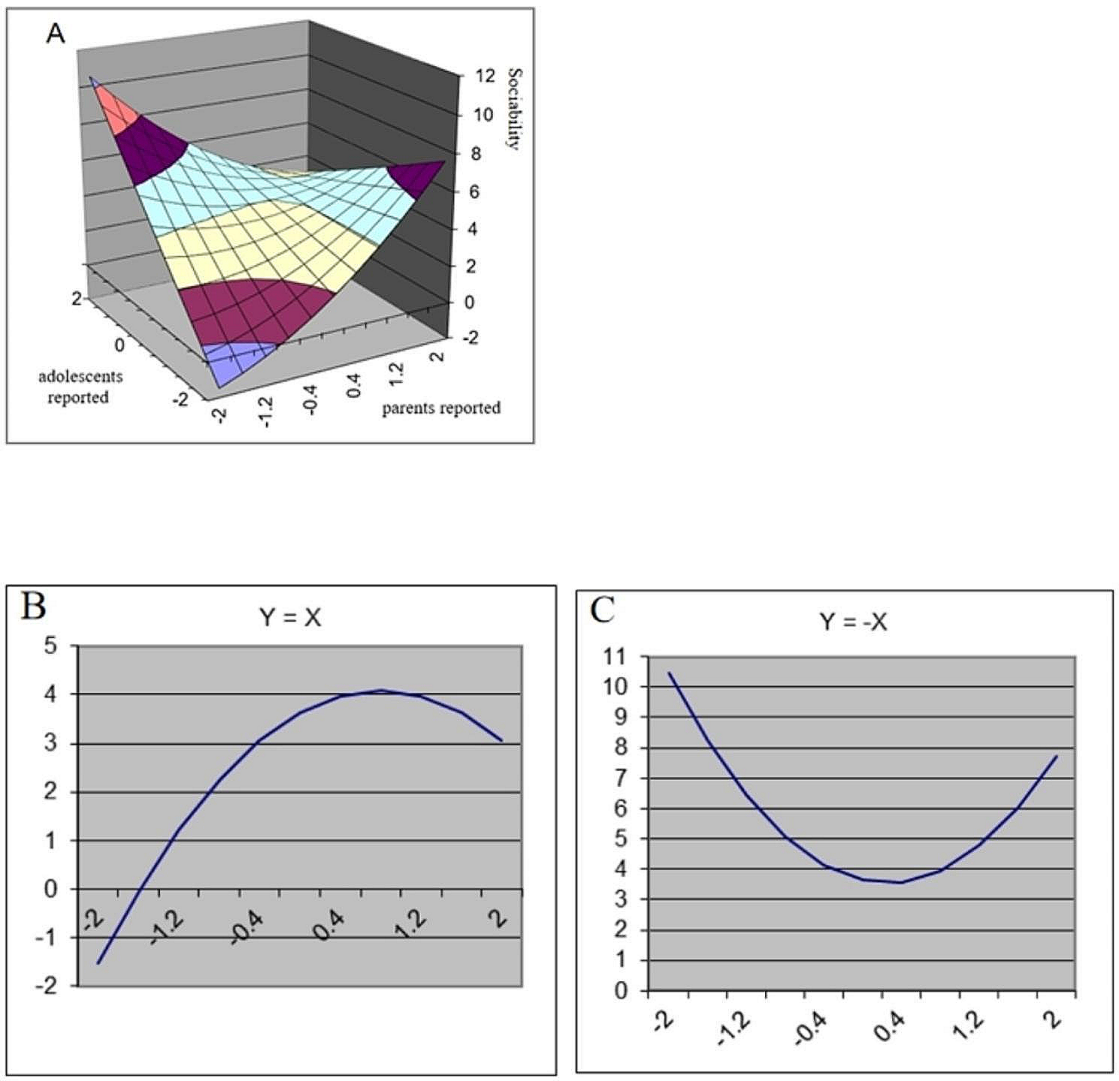
Response Surface Analysis plots for adolescents’ sociability

**Fig. 4:**
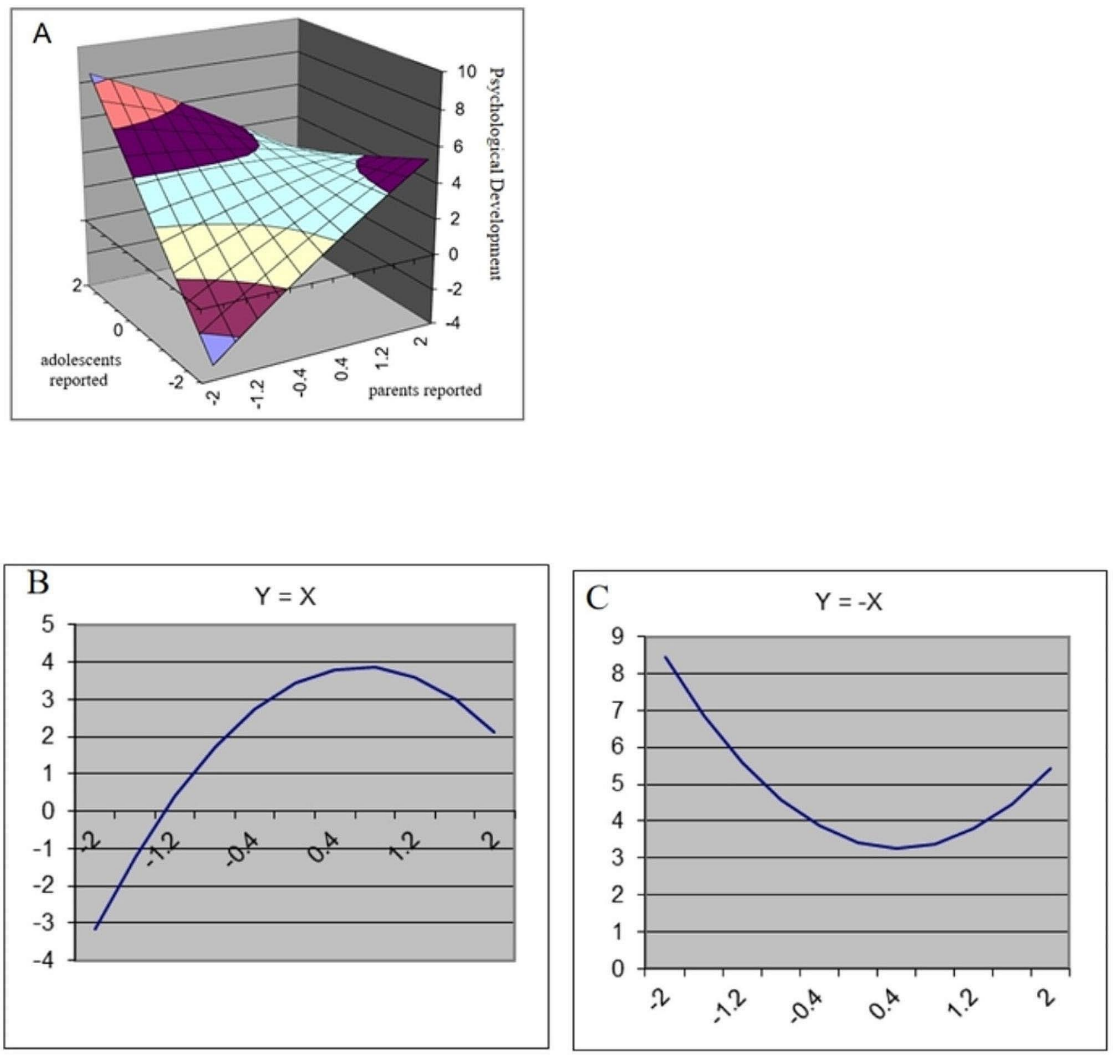
Response Surface Analysis plots for adolescents’ psychological development

The results of the polynomial regression analyses and response surface analyses are presented in Table 2. In addition, Figs. 1, 2, 3 and 4 show a visualization of the response surface results (coping ability/self-improvement/sociability/psychological development).

*Coping Ability*. As shown in Table 3, adolescents’ reported family resilience was significantly associated with their coping ability (b2 = 0.87, *p* < 0.001), whereas the effect of parent-reported family resilience was not significant. The squared terms reported by adolescents and parents were not significant, but their interaction terms were significant (b4=-0.78, *p* < 0.05). As shown in Fig. 1, LOC had a significant linear effect (a1 = 1.09, *p* < 0.001) and a curvilinear effect (a2=-1.23, *p* < 0.01). The significantly positive a1 suggested that high levels of congruent family resilience were associated with higher coping ability in adolescents than were low levels of congruence. However, the significantly negative a2 reveals that from some point, when both adolescents and their parents reported increased family resilience, the adolescent’s coping ability decreased (Fig. 1B). In addition, the significant negative linear effect (a3=-0.65, *p* < 0.01) and nonsignificant curvilinear effect of LOIC indicated that higher levels of coping ability were reported by adolescents when adolescents reported higher family resilience compared to their parents (Fig. 1C).

**Table 3:** Adolescent–parent congruence and incongruence as predictors of adolescents’ psychological adjustment

	Coping Ability	Self-improvement	Sociability	Psychological Development
Polynomial regression coefficient				
b_0_ (SE)	3.53^***^(0.06)	3.58^***^(0.06)	3.64^***^(0.06)	3.43^***^(0.06)
b_1_-Adolescent report(SE)	0.22(0.17)	0.52^**^(0.16)	0.23(0.17)	0.29(0.18)
b_2_-Parent report (SE)	0.87^***^(0.12)	0.97^***^(0.12)	0.92^***^(0.12)	1.04^***^(0.13)
b_3_-Adolescent report^2^ (SE)	-0.68(0.27)	-0.50(0.26)	0.31(0.26)	-0.08(0.29)
b_4_-Adolescent*Parent report (SE)	-0.78^*^(0.37)	-0.95^**^(0.36)	-1.04^**^(0.36)	-0.93^*^(0.39)
b_5_-Parent report^2^ (SE)	0.23(0.24)	0.07(0.23)	0.02(0.24)	0.02(0.26)
Response surface parameters				
a1(SE)	1.09^***^(0.19)	1.49^***^(0.18)	1.15^***^(0.19)	1.60^***^(0.20)
a2(SE)	-1.23^**^(0.37)	-1.38^***^(0.35)	-0.72*(0.36)	-0.99*(0.39)
a3(SE)	-0.65^**^(0.23)	-0.45^*^(0.22)	-0.69^**^(0.22)	-1.02^***^(0.24)
a4(SE)	0.33(0.50)	0.52(0.48)	1.36^**^(0.49)	0.88(0.53)

SE, standard error; a1, line of congruence; a2, curvilinearity in line of congruence; a3, line of incongruence; a4, curvilinearity in line of incongruence

*p < 0.05, **p < 0.01, ***p < 0.001

*Self-improvement*. As shown in Table 3, parent-reported family resilience (b1 = 0.52, *p* < 0.01), adolescent-reported family resilience (b2 = 0.97, *p* < 0.001), and the parent-adolescent interaction term (b4=-0.95, *p* < 0.01) were significantly related to adolescents’ self-improvement. As shown in Fig. 2, the LOC had a significant linear effect (a1 = 1.49, *p* < 0.001) and curvilinear effect (a2=-1.38, *p* < 0.001). The significantly positive a1 suggested that high levels of congruent family resilience were associated with higher self-improvement in adolescents. However, the significantly negative a2 reveals that this effect was not linear (Fig. 2B). In addition, the significant negative linear effect (a3=-0.45, *p* < 0.05) and nonsignificant curvilinear effect of LOIC indicated that when adolescents reported higher family resilience than their parents, adolescents showed more self-improvement (Fig. 2C).

*Sociability*. Polynomial regression analyses revealed that adolescent-reported family resilience (b2 = 0.92, *p* < 0.001) and the adolescent-parent interaction term (b4=-1.04, *p* < 0.01) were significantly related to adolescents’ sociability. As shown in Fig. 3, the LOC had a significant linear effect (a1 = 1.15, *p* < 0.001) and curvilinear effect (a2=-0.72, *p* < 0.05). These findings suggest that high levels of congruent family resilience were associated with higher sociability in adolescents, and this effect was not linear (Fig. 3B). In addition, the LOIC had a significant linear effect (a3=-0.69, *p* < 0.01) and curvilinear effect (a4 = 1.36, *p* < 0.01). The significantly negative a3 suggested that when adolescents reported higher family resilience than their parents, adolescents showed more sociability. The significantly positive a4 indicated that adolescents’ sociability declined when the family resilience reported by adolescents and their parents converge (Fig. 3C).

*Psychological Development*. As shown in Table 3, adolescent-reported family resilience (b2 = 1.04, *p* < 0.001) and the parent-adolescent interaction term (b4=-0.93, *p* < 0.05) were significantly related to adolescents’ psychological development. As shown in Fig. 4, LOC had a significant linear effect (a1 = 1.60, *p* < 0.001) and a curvilinear effect (a2=-0.99, *p* < 0.01), suggesting that high levels of congruent family resilience were associated with higher psychological development in adolescents and that this effect was not linear (Fig. 4B). In addition, the significant negative linear effect (a3=-1.02, *p* < 0.001) and nonsignificant curvilinear effect of LOIC indicated that higher levels of psychological development were reported by adolescents when adolescents reported higher family resilience compared to their parents (Fig. 1C).

## Discussion

This study examined how the discrepancies in perceived family resilience between parents and adolescents with chronic illness were associated with adolescents’ psychological adjustment. In the present study, we found that adolescents’ reported family resilience was not diverse in terms of demographic characteristics and disease type, whereas parent-reported family resilience differed significantly in adolescent gender, parents’ religious beliefs, and education level. As girls are more submissive and dependent, and closer to their parents [25], parents may experienced more closer family relationships, resulting in higher family resilience. In contrast to most previous studies [48], parents with no religious belief reported higher family resilience in this study. This may be because most Chinese parents do not believe in any religion, and only those who are unable to find resources within or outside the family will turn to religion for help [45]. Moreover, parents with higher education levels are better equipped with information related to disease treatment [49], thus finding more family resilience in the adversity of adolescent chronic illness.

Our study found that both parent- and adolescent-reported family resilience were positively associated with the psychological adjustment of adolescents with chronic illness. Families with high levels of resilience are characterized by strong self-efficacy, empathy, and family relationships during the treatment process [50], which can improve their ability to cope with illness and improve their mental health [51]. Our results revealed that adolescent-reported family resilience had a stronger correlation with adolescents’ psychological adjustment than parent-reported family resilience. These results support the interdependence theory of actor-partner effect theory; that is, the actor effect is more significant with respect to the outcome variable than the partner effect [52, 53]. The results of this study are similar to the findings of Human [19], which state that adolescents’ perception of the family environment was more strongly correlated with their psychological adjustment than their parents’. Adolescents is characterized by characteristics such as less dependence on adults and becoming independent from parents [54], therefore they may be more concerned with their own thoughts and may neglect their parents.

This finding is consistent with Hypothesis one. Our findings also echoed the operations triad model by demonstrating that parent-adolescent convergence of a high level of family resilience improved the psychological adjustment of children with chronic illness, which is consistent with the results of previous studies [20, 32]. According to the structural family theory [55], parent-child interactions jointly influence children’s developmental outcomes. Hence, the convergent perception of family resilience between parents and adolescents implies good family communication and effective coping abilities in the face of the adversity associated with illness [30], thereby decreasing the likelihood of adolescents being affected by the illness and increasing the level of psychological adjustment. Interestingly, not all high levels of family resilience consistency were favorable; from some point on, adolescents’ psychological adjustment decreased when both adolescents and their parents reported a relatively higher level of family resilience. This suggests a lack of individualization between adolescents and their parents [19]. Therefore, future studies are required to examine the mechanisms through which adolescent- and parent-reported family resilience affect adolescent psychological adjustment, particularly considering the level of family resilience.

There is a high degree of discrepancy in family resilience between parents and adolescents with chronic illness, with adolescents reporting a higher level of family resilience than their parents. There are several possible explanations for these results. First, as Chinese culture emphasizes emotional restraint [15], one’s inner thoughts are often preserved, and discussing family crises is viewed as poor adjustment [56]. Therefore, parents often choose not to disclose family difficulties to their children [57]. Moreover, since the diagnosis of childhood chronic illness, parents spend more time accompanying their children and become more attentive to the adolescent’s physical health [58]. Therefore, adolescents reported higher levels of family resilience. In addition, adolescents are more adaptable and flexible than their parents and can adapt more quickly to the effects of the disease. For example, parents think that illness leads to internal family conflict, family dysfunction, and a lack of family support [59]. In contrast, adolescents think that illness leads to inner growth and a better way to face the future [60]. As a result, adolescents with chronic illness perceive higher family resilience than their parents.

Importantly, consistent with our second hypothesis, our study found adolescents had lower psychological adjustment (coping ability, self-improvement, and psychological development) when parents reported family resilience exceeded that of adolescents, and vice versa. Such findings indicated that the active reports of adolescents about the family environment (compared to their parents) represent better adjustment, while the negative reports of adolescents about the family environment (compared to their parents) represent psychological maladjustment [61]. Unlike previous studies that analyzed discrepancies in perceived family functioning between normal adolescents and their parents [19, 30], the primary study population of the current study was adolescents with chronic illness, whose parents spend a large amount of time, energy, and financial resources raising and caring for their children [24]. When parents overestimate the level of family resilience due to their dedication, they are prone to under-recognize the true intrinsic crisis in the family and ignore the true emotional needs of adolescents with chronic illness [61]. When parents fail to recognize their children’s real emotions, adolescents may choose to avoid stressful communication and restrain the expression of thoughts and feelings, causing parents to further disregard the child’s true inner emotions [62], ultimately leading to adolescent maladjustment. In contrast, adolescents’ evaluations of family resilience reflect their true inner thoughts and feelings. Therefore, helping adolescents to develop open and transparent communication about stress with their parents may be an effective measure to improve psychological adjustment.

Notably, there were significant linear and curvilinear effects on adolescents’ sociability, indicating an association between adolescents’ sociability and discrepancy in family resilience reported by parents and adolescents. Interestingly, our results showed that adolescents’ sociability decreased as adolescent- and parent-reported family resilience converged. Unsurprisingly, adolescents may have an adverse psychological condition when both themselves and their parents report low family cohesion and resilience [63]. However, whether parents and adolescents consistently perceive lower family resilience when they are less sociable remains unknown. Future studies should further explore the mechanisms between family resilience and sociability using more statistical analytical techniques.

### Limitations and future directions

This study has several limitations. First, because this is a cross-sectional study, a causal relationship between the study variables could not be established. The psychological adjustment of adolescents with chronic illness can change over time [60]. Future longitudinal studies are needed to predict whether discrepancies in perceived family resilience among adolescents with chronic illness and their parents can predict the trajectory of adolescent psychological adjustment.

Secondly, because of the Chinese culture of “male domination of the outside world and female domination of the inside world“ [64], the majority of participants in our sample were mothers. Family systems theory argues that adolescent-mother and adolescent-father dyads represent distinct but related subsystems [65]. Future studies should take the parents’ gender into consideration when studying the discrepancy between parent-adolescent dyads to gain a more complete picture and analyze differences in perceived family resilience between “father-adolescents” and “mother-adolescents,” as well as the relationship to adolescent psychological adjustment.

In addition, this study uses convenience sampling, with a relatively small sample drawn from three hospitals in eastern China, making the sample less representative. To generalize the findings of this study, future research should involve a more diverse and larger sample from different geographical regions.

The present study focused only on positive internalizing behaviors in adolescents. Different patterns of negative emotions and externalizing behaviors may emerge in adolescents [66]. Further expansion of relevant research is needed to explore the relationship between discrepancies in perceived family resilience and adolescent negative emotions among adolescents and their parents, adding to the completeness and rigor of the research.

Finally, it is unclear whether the findings that adolescent-reported family resilience had a stronger correlation with adolescents’ psychological adjustment than those reported by parents are correct or whether these associations are simply due to observer bias. This is an important question for future research, and alternative methods are required. For example, obtaining and citing reports from third parties (e.g., siblings) is feasible and can eliminate bias.

### Clinical implication

These findings have several practical implications. The results of the current study show that convergence in adolescent- and parent-reported family resilience is positively associated with adolescents’ psychological adjustment, and parents’ overestimation of family resilience negatively affects their level of psychological adjustment; therefore, strategies to promote the convergent perception of family resilience in parent-adolescent dyads are warranted. A systematic review indicated that parent-child communication is directly related to family resilience and cohesion, family relationships, family satisfaction, and family problem-solving skills [67]. Therefore, parents should openly communicate with their adolescents about their illness and family situation to improve their level of psychological adjustment. Currently, studies have been conducted to improve the psychological adjustment of adolescents with behavioral problems by improving the quality of parent-child communication [68]. Future interventions need to be tailored to promote parent-child communication among adolescents with chronic illness.

## Conclusion

This study used polynomial regression and response surface analysis to explore the relationship between discrepancies in parent- and adolescent-reported family resilience and the psychological adjustment of adolescents with chronic illness. Our results showed that adolescent-reported family resilience had a stronger correlation with adolescents’ psychological adjustment. Both convergent and divergent family resilience between parent and adolescent dyads are significantly associated with adolescents’ psychological adjustment. Parents’ overestimation of family resilience indicates lower levels of psychological adjustment. This study suggests a targeted intervention to improve parent-child communication to reduce the discrepancy in perceived family resilience among parent-adolescent dyads, which may benefit the mental health of adolescents with chronic illnesses.

## Data Availability

Due to the involvement of participant privacy and confidentiality agreement constraints, the dataset generated during the current research period is not publicly available, but can be obtained from the corresponding author upon reasonable request.

## Abbreviations

RSA: response surface analysis
LOC: line of congruence
LOIC: the line of incongruence
