# Correction: Discrepancies in perceived family resilience between adolescents with chronic illness and parents: using response surface analysis to examine the relationship with adolescents’ psychological adjustment

**DOI:** 10.1186/s12888-024-05944-4

**Published:** 2024-07-12

**Authors:** Meijia Chen, Liya Ren, Hao Jiang, Yuxin Wang, Liping Zhang, Chaoqun Dong

**Affiliations:** 1 The Second Affiliated Hospital and Yuying Children’s Hospital of Wenzhou Medical University, Wenzhou, 325027 China; 2 School of Nursing, Wenzhou Medical University, University Town, Chashan, Wenzhou, 325035 China; 3 Clinical Skills Center, The Second Affiliated Hospital and Yuying Children’s Hospital of Wenzhou Medical University, 109 Xueyuan West Road, Wenzhou, 325027 China

**Chen et al. BMC Psychiatry (2024) 24:475**

10.1186/s12888-024-05917-7

Following publication of the original article [1], the authors identified that Chaoqun Dong was incorrectly assigned to affiliation 3. The correct affiliation is now assigned to Chaoqun Dong.

The incorrect affiliation of Chaoqun Dong is:

^3^linical Skills Center, The Second Affiliated Hospital and Yuying Children’s Hospital of Wenzhou Medical University, 109 Xueyuan West Road, Wenzhou 325,027, China.

The correct affiliation of Chaoqun Dong is:

^2^School of Nursing, Wenzhou Medical University, University Town, Chashan, Wenzhou 325,035, China.

The original article [1] has been corrected.

## References

[1] Chen et al. BMC Psychiatry (2024) Discrepancies in perceived family resilience between adolescents with chronic illness and parents: using response surface analysis to examine the relationship with adolescents’ psychological adjustment (2024) 24:475. 10.1186/s12888-024-05917-7 10.1186/s12888-024-05917-7 PMC11210177 38937737

